# Carcinoma cells misuse the host tissue damage response to invade the brain

**DOI:** 10.1002/glia.22518

**Published:** 2013-07-06

**Authors:** Han-Ning Chuang, Denise van Rossum, Dirk Sieger, Laila Siam, Florian Klemm, Annalen Bleckmann, Michaela Bayerlová, Katja Farhat, Jörg Scheffel, Matthias Schulz, Faramarz Dehghani, Christine Stadelmann, Uwe-Karsten Hanisch, Claudia Binder, Tobias Pukrop

**Affiliations:** 1 Department of Hematology/Oncology, University Medical CenterGöttingen, Germany; 2 Institute of Neuropathology, University Medical CenterGöttingen, Germany; 3 EMBL HeidelbergMeyerhofstraße 1, Heidelberg, Germany; 4 Department of Neurosurgery, University Medical CenterGöttingen, Germany; 5 Department of Medical Statistics, University Medical CenterGöttingen, Germany; 6 Department of Cardiovascular Physiology, University Medical CenterGöttingen, Germany; 7 Institute of Anatomy, University LeipzigLeipzig, Germany

**Keywords:** astrocytes, brain metastasis, damage response, glia, invasion, microglia

## Abstract

The metastatic colonization of the brain by carcinoma cells is still barely understood, in particular when considering interactions with the host tissue. The colonization comes with a substantial destruction of the surrounding host tissue. This leads to activation of damage responses by resident innate immune cells to protect, repair, and organize the wound healing, but may distract from tumoricidal actions. We recently demonstrated that microglia, innate immune cells of the CNS, assist carcinoma cell invasion. Here we report that this is a fatal side effect of a physiological damage response of the brain tissue. In a brain slice coculture model, contact with both benign and malignant epithelial cells induced a response by microglia and astrocytes comparable to that seen at the interface of human cerebral metastases. While the glial damage response intended to protect the brain from intrusion of benign epithelial cells by inducing apoptosis, it proved ineffective against various malignant cell types. They did not undergo apoptosis and actually exploited the local tissue reaction to invade instead. Gene expression and functional analyses revealed that the C-X-C chemokine receptor type 4 (CXCR4) and WNT signaling were involved in this process. Furthermore, CXCR4-regulated microglia were recruited to sites of brain injury in a zebrafish model and CXCR4 was expressed in human stroke patients, suggesting a conserved role in damage responses to various types of brain injuries. Together, our findings point to a detrimental misuse of the glial damage response program by carcinoma cells resistant to glia-induced apoptosis.

## Introduction

Brain metastasis represents a severe clinical problem. In particular, carcinomas of the lung and breast metastasize very frequently to the brain with tremendous impact on overall survival rate (Eichler et al., 11). We recently demonstrated that there is an overall survival rate of only 7 months after neurosurgical resection for lung adenocarcinoma patients (Bleckmann et al., 6). In advanced breast cancer, 30% of the patients develop brain metastasis. Interestingly, cerebral metastasized breast cancer patients without neurological symptoms have the same unfavorable prognosis as patients with symptomatic brain metastasis. More importantly, these patients have a worse prognosis in comparison to patients with metastases at other sites, i.e., bone, liver, or lung (Miller et al., 31). This difference may result from the special characteristics of the brain tissue and its unique microenvironment (Eichler et al., 11; Fidler, 12; Steeg et al., 47).

Interactions between malignant cells and the microenvironment play a pivotal role in the first steps of metastasis. In particular, in the primary tumor tumor-associated macrophages (TAM) can support cancer cell proliferation, migration, invasion, and intravasation as well as participate in matrix remodeling and angiogenesis (Allavena et al., 1; Balkwill et al., 2; Mantovani et al., 30). These tumor-promoting features in the primary are reflected in the clinical outcome (Bingle et al., 5). Interestingly, the typical target organs of metastasis possess tissue-specific resident macrophages, such as Kupffer cells in the liver, alveolar macrophages in the lung, or microglia in the central nervous system (CNS).

Microglia are important in the maintenance and protection of CNS homeostasis and functionality (Hanisch and Kettenmann, 17). They constitute the first line of defense and exert effector functions on various infections and other kinds of brain damage (e.g. stroke and brain injuries). Interestingly, microglia express a range of pattern recognition receptors (PRRs), including Toll-like receptors (TLRs), which allow the detection of both infection as well as damage signals (Kettenmann et al., 20). While pathogen-associated molecular patterns (PAMPs) refer to an assortment of evolutionary conserved structural motifs in glyco- and lipopeptides, glycolipids, DNA and RNA of bacteria, viruses, fungi, or protozoa (Kawai and Akira, 19; Takeuchi and Akira, 49), danger-associated molecular patterns (DAMPs) comprise a collection of disparate molecules of endogenous origin. Under normal conditions they have diverse functions, but acquire a danger signal upon some nonphysiological release and/or structural modification (Rubartelli and Lotze, 40). They can drive a tissue response also in the absence of infection agents, i.e. best described as sterile inflammation (Chen and Nunez, 10). DAMPs may prepare, support, or modify reactions to PAMPs and install programs for wound healing and repair. However, in certain pathological settings, they do participate in destructive processes and even self-propelling cycles (Lehnardt et al., 27; Stewart et al., 48). Their PRR/TLR equipment renders microglia potentially sensitive to a plethora of DAMPs, including those of cellular (heat shock proteins, high-mobility group protein B1, and S100 calcium binding protein A8/A9), plasma, or extracellular matrix (ECM) origin (fibrinogen, fibronectin, tenascin, and versican). Furthermore, microglia are not simply derivatives from monocytic cells of the bone marrow. They develop from yolk sac progenitors, maintain themselves as a stable population by self-renewal independently of the hematopoiesis and possess the capacity to proliferate on demand (Ginhoux et al., 14; Kierdorf et al., 2013; Schulz et al., 44). For example, infections or injuries lead to an activation and proliferation of microglia. Most importantly, activation of microglia primarily serves in CNS protection but can occasionally contribute to destructive processes and thereby aggravate clinical symptoms upon acute insults or in chronic (neurodegenerative) diseases (Hanisch and Kettenmann, 17; Kettenmann et al., 20; Prinz et al., 36).

However, in contrast to the role of bone marrow-derived TAM in the primary tumor, the functions of these tissue-specific macrophage-like cells during metastatic colonization and the subsequent tissue damage of the respective organs have barely been investigated, in the case of microglia they have been almost ignored (Gjoen et al., 15; Heuff et al., 18; Steeg et al., 47). Nevertheless, there are few histological descriptions clearly demonstrating microglia-carcinoma cell interactions. In particular, they revealed that microglia infiltrate into the tumor mass, accumulate in the surrounding gliosis zone, and create contacts with the carcinoma cells directly after successful extravasation (Fitzgerald et al., 13; Lorger and Felding-Habermann, 28; Zhang and Olsson, 52). These observations not only suggest a role of microglia during brain metastasis but also reveal that they interact directly after carcinoma cell extravasation. Knowing that extravasation alone is not sufficient for successful metastatic growth in the liver as well as brain and that the majority of the carcinoma cells undergo apoptosis afterward, it makes the microglia-carcinoma cell interaction at this time point even more interesting (Chambers et al., 9; Kienast et al., 21). Reasons for the lack of reports on glial-carcinoma interactions relate to the difficulty of distinguishing resident microglia from invading bone marrow-derived monocytes/macrophages using histology. It is also due to the limited availability of patient samples, and the absence of suitable mouse models to study the brain microenvironment during cerebral colonization.

We therefore established an *ex vivo* coculture model, which consists of an organotypic brain slice and a 3D-carcinoma cell plug. This method allows not only the direct visualization of carcinoma cell-microglia interactions without any contamination of blood-derived macrophages but also allows us to study and target microglia as well as other resident cells within the brain tissue. In direct contrast to the expected tumor defense function, microglia in this coculture model fostered malignant invasion of different breast cancer cell lines (MCF-7 and 410.4) by serving either as guiding structures or even as transporters for single invading carcinoma cells as well as carcinoma cell cohorts (Pukrop et al., 37). Interestingly, gene expression studies of inflammatory microglia in coculture with carcinoma cells identified TLR and WNT signaling as the most affected pathways in microglia. TLR signaling is by far the best described system for DAMP actions, and WNT signaling controls very important functions in tissue regeneration and repair. Moreover, WNT pathways are essential for the communication between TAM and carcinoma cells (Ojalvo et al., 34; Pukrop et al., 38) as well as in the formation of brain metastasis (Bleckmann et al., 6; Klemm et al., 25; Nguyen et al., 33; Smid et al., 46).

In addition, the C-X-C chemokine receptor 4 (CXCR4), recently described as a DAMP receptor for extracellular ubiquitin (Saini et al., 41), was identified as the most upregulated gene in microglia (Pukrop et al., 37). CXCR4 has been characterized in different cancer types as a progression marker, its inhibition preventing metastasis formation (Zlotnik et al., 53). Its chemokine ligand stroma-derived factor 1 (SDF1) has also been found to be massively upregulated after various brain injuries, thus indicating a role in benign brain damage responses (Bye et al., 8).

In our current study, we employed our brain slice coculture system, a transgene zebrafish model as well as human brain metastasis samples to further dissect molecular elements and cellular mechanisms of the recently described unexpected phenomenon of microglia-assisted invasion. Furthermore, our aim was to determine (i) the specificity of this process for carcinoma cells, (ii) the potential involvement of cells other than microglia, and (iii) the role of CXCR4 as well as (iv) to investigate essential WNT signaling contributions during this process in more detail.

## Materials and Methods

### Cell Lines and Primary Culture

The human breast cancer cell line MCF-7 and the Madin–Darby canine kidney cell line (MDCK) were purchased from DSMZ and ATCC, respectively. They were maintained in RPMI-1640 medium (PAA Laboratories, Cölbe, Germany) supplemented with 10% fetal calf serum (FCS, Invitrogen, Karlsruhe, Germany). The human mammary epithelial cell line (hTERT-HME1) was purchased from BD Biosciences Clontech (Heidelberg, Germany) and was maintained in mammary epithelial cell medium (PromoCell, Heidelberg, Germany). Cells were mycoplasma-free during regular controls. Stable transfections with the mammalian Turbo GFP vector (FP512, Evrogen, Heidelberg, Germany) were performed with the Nanofectin kit (PAA Laboratories, Cölbe, Germany) in accordance with the manufacturer’s protocol and selection was performed through geneticin resistance (G418, Roche, Basel, Switzerland). To obtain homogeneous GFP expression, cells were sorted using fluorescence-activated cell sorting method (FACS, BD FACS Aria II, Heidelberg, Germany). Primary microglia and astrocytes from newborn mice were prepared as previously described (Regen et al., 39). Cell viability was measured by MTT conversion according to standard protocols.

### Microinvasion Assay and Organotypic Brain Slice Coculture System

The degree of invasion of MCF-7 and MDCK cells was measured in coculture with astrocytes or microglia without direct cell-to-cell contact using a modified Boyden chamber assay as described previously (Pukrop et al., 37). 10^5^ MCF-7 or MDCK cells and 2 × 10^5^ of microglia or astrocytes were applied for 96 h. Mice of the C57BL/6 or NMRI were decapitated between postnatal day 6 and 8 for the organotypic brain slice coculture experiments, which were performed with the interface technique following the previously described protocol (Kreutz et al., 26; Pukrop et al., 37). To investigate the influence of different cortex regions, brain slices were categorized into three levels according to the anatomical planes. As means of orientation, we used the architecture of the cerebrum, brain stem, and ventricles in the obtained slices. We obtained six horizontal whole brain slices in general. The first two cranial slices were defined as level one, the third and fourth were level two, and both caudal slices as level three. These organotypic brain slices were cocultured with 10^5^ MCF-7 or MDCK cells, embedded in 85% extracellular matrix (ECM gel; R&D Systems, Wiesbaden, Germany). Recombinant Dickkopf 2 (DKK2, 200 ng/mL, R&D systems, Wiesbaden, Germany) and AMD3100 octahydrochloride (AMD3100, also known as plerixafor, 1 µg/mL, Sigma, Munich, Germany) were applied to inhibit the WNT and CXCR4 pathway, respectively. The time-lapse imaging experiments were performed on a Leica inverted DMI 6000B microscope with a 10-fold magnification lens and a Leica DFC 350 FX CCD camera (Leica, Wetzlar, Germany). As recently described, the live staining of microglia was performed with 2 µg ILB4-Alexa Fluor 568 in advance. Images were processed using the software Video Spin (http://www.videospin.com/Redesign/).

### Histology and Immunohistochemistry of Brain Slices and Human Tissues

Human tumor tissue samples were obtained from neurosurgically resected brain metastases following approval of the local ethics committee. Immunohistochemistry was performed based on a modification of a previously described protocol (Nessler et al., 32). The KiM1P staining was performed as previously described (Pukrop et al., 37). Human tissue and brain slice coculture sections for immunohistochemistry were embedded in paraffin, cut into 2–5 µm sections with a microtome (Leica SM 2000R, Leica, Wetzlar, Germany) and mounted on glass slides. The sections were de-waxed, rehydrated, and stained with the following primary antibodies: polyclonal rabbit anti-CXCR4 protein (1:100, Abcam, Cambridge, UK), monoclonal anti-KiM1P antibody (1:500, kindly provided by Prof. H. J. Radzun, Göttingen, Germany), and polyclonal rabbit anti-glial fibrillary acidic protein (GFAP) (1:200, Dako, Glostrup, Denmark) to detect astrocytes. Pretreatment with 10 mM citrate buffer (pH 6.0) in a microwave oven for 3 min was required in preparation for GFAP staining. Antibody detection was achieved with biotinylated anti-rabbit secondary antibody followed by avidin-peroxidase. Aminoethylcarbazole (AEC) was used as chromogen for CXCR4 staining. Control sections were incubated either in absence of primary antibodies or with isotype controls or with nonimmune sera. After counterstaining with hematoxylin, slides were covered with coverslips and analyzed. To quantify the CXCR4 expression in human tissue, we evaluated the slides with a microscope according to the following scoring system: 0 = none of the cells; + = up to 5%; ++ = 5–10%; and +++ = >10% express CXCR4.

### Immunofluorescence Staining of Astrocytes and Microglia in the Organotypic Brain Slice Coculture

Immunofluorescence staining of microglia and counterstaining with DAPI were completed as previously described (Pukrop et al., 37). Astrocytes were detected by anti-GFAP (1:200, Sigma, Munich, Germany). Organotypic cocultures were incubated with the primary anti-GFAP antibody for 36 h at 4°C, followed by anti-mouse-TRITC staining for 1 h (1:100, Sigma, Munich, Germany). The degree of invasion was evaluated using the recently described scoring system from 0 to +++ (Pukrop et al., 37). The degree of microglia accumulation at the interface to the tumor plug was categorized with following scoring system: 1+ = <25%; 2+ = 25–50%; 3+ = 50–75%; and 4+ = ≥75% of the contact area with the cell plug of the whole brain slice surface was positive for microglia. Double staining of microglia and astrocytes was performed using anti-GFAP and anti-mouse-TRITC antibodies as described above, followed by ILB4-Alexa Fluor 647 (Invitrogen, Germany) and DAPI counterstaining. Samples were mounted in DAKO fluorescent mounting medium (Dako, Glostrup, Denmark), coverslipped, and analyzed with a Zeiss confocal laser scanning microscope (LSM 510, Zeiss, Göttingen, Germany).

### Live–Dead Staining

MCF-7 and MDCK cells were cocultured with brain slices for up to 96 h. To investigate the related mechanism, bisphosphonate clodronate (100 µg/mL, Roche, Basel, Switzerland), DKK2 (200 ng/mL), and AMD3100 (1 µg/mL) were applied. Live and dead cells were quantified using a Calcein-AM and propidium iodide (PI) double staining kit according to the manufacturer’s protocol (AnaSpec, CA). Calcein-AM and PI-positive cells were counted in three random fields with the Image J software (Image J 1.43f). Results were described as a percentage of the numbers of living cells to total cells (live cells/live cells + dead cells). The UV-irradiated samples served as positive control.

### Reverse Transcription and Quantitative Real-Time Polymerase Chain Reaction

Total RNA was isolated using the TRIzol Reagent (Invitrogen, Karlsruhe, Germany) including a DNase I digestion. cDNA synthesis and PCR were performed as previously described (Klemm et al., 25). Whole brain slices were treated with clodronate (100 µg/mL) for 48 h. For primer sequences, please refer to Supporting Information Table 1. Gene expression levels were normalized to the average of the respective housekeeping (HK) genes and expressed as fold changes (fc) followed by analyses with the comparative cycle threshold (Ct) method as described (Pukrop et al., 37).

**TABLE 1:** CXCR4 Expression in Cerebral Brain Metastasis of Breast Cancer and Lung Cancer Samples

		CXCR4 expression in the carcinoma cells					CXCR4 expression in the adjacent gliosis			
Tissue type	Total amount	0	+	++	+++	Total amount	0	+	++	+++
Breast cancer	23	3 (13.0%)	12 (52.2%)	6 (26.1%)	2 (8.7%)	15	3 (20.0%)	7 (46.7%)	4 (26.7%)	1 (6.6%)
Lung cancer	22	5 (22.7%)	13 (59.1%)	4 (18.2%)	0 (0%)	16	5 (31.2%)	8 (50%)	3 (18.8%)	0 (0%)

Human patient samples stained for CXCR4 with IHC in normal brain, lung, and breast cerebral metastasis samples. The metastasis samples were analyzed for CXCR4 expression in the carcinoma cells as well as in the gliosis zone if available.

### Immunoblot (Western Blot) and Immunofluorescence Staining for CXCR4

To assess protein expression and the distribution of CXCR4 on different cells, we performed immune-blot (IB) and immunohistochemical staining with the primary rabbit polyclonal CXCR4 antibody (1:2,000, Abcam, Cambridge, UK). To prepare for IB, samples of whole cell lysate from MCF-7, MDCK, primary microglia or astrocytes (40 μg each) were processed with 10% SDS-polyacrylamide gel (PAGE) and visualized using enhanced chemoluminescence (ECL, GE Healthcare, Pollards Wood, UK) with the Fuji LAS 4000 image system. For the immunofluorescence, cells were incubated with the primary antibody for 1 h at RT (1:100, Abcam, Cambridge, UK), followed by staining with anti-rabbit-TRITC for 1 h (1:100, Sigma, Munich, Germany). They were then counterstained with DAPI, coverslipped, and analyzed with a confocal microscope as described above.

### Analysis of Microarray Datasets

Three Affymetrix datasets containing 28 brain metastasis samples of primary adenocarcinomas of the lung (GSE14108), 22 brain metastases from primary breast cancers (GSE14017, GSE14018), and 11 brain tissue samples of healthy controls (GSE5389) were retrieved from the NCBI Gene Expression Omnibus (GEO) data repository (Barrett and Edgar, 3). All samples were combined into one dataset and quantiles normalized. All analyses were performed using the free statistical software R (version 2.15.1; http://www.r-project.org).

### Microglial Chemotaxis Assay

Microglia were seeded into inserts (3 µm pore size, BD Biosciences, Heidelberg, Germany) at a density of 0.7 × 10^5^/300 µL culture medium and pretreated after 1 h with 1 µg/mL AMD3100 for 2 h. Microglia-containing inserts were placed into 24-well dishes into which either 0.7 × 10^5^ MCF-7 cells or MDCK cells had been seeded before and stained with 2 mM Calcein-AM (Sigma-Aldrich, Munich, Germany). Nonmigrated cells were removed from the upper side of the insert membrane by intense scraping, while migrated microglia on the lower side were fixed with 4% PFA and analyzed using fluorescence microscopy (Axio Observer D1, Carl Zeiss, Göttingen, Germany).

### Transgenic Zebrafish Experiments

Fish were kept at 26.5°C in a 14 h light/10 h dark cycle. Embryos were collected by natural spawning and raised at 28.5°C in E3-solution. To avoid pigmentation, 0.003% PTU was added 1 day postfertilization (dpf). Embryos were staged according to Kimmel et al. (24). Microglia were visualized using the pu1:Gal4-UAS-GFP transgenic line (Peri and Nusslein-Volhard, 35). For live imaging purposes, three dpf zebrafish larvae were anesthetized in 0.01% tricaine and embedded in 1.5% low melting point agarose. We used an Olympus FV 1000 with a 40X/NA1.15 objective for the imaging. In general, we captured 30–40 *z*-stacks spanning 45–60 µM for full brain recordings. Images were flattened by maximum projection in Imaris (Bitplane) and Fiji software. Lesions were generated using a pulsed 532 nm laser coupled to the FV 1000. Images were analyzed using both Imaris (Bitplane) and Fiji software. The CXCR4 morpholino oligonucleotide (5′-AAATGATGCTATCGTAAAATTCCAT-3′) was obtained from Gene Tools. The morpholino was injected at a concentration of 1 mM with 0.2% phenol red (Sigma) and 0.1 M KCl (Sigma) into one-cell-stage zebrafish embryos.

### Statistics

All data represent the mean ± standard error of the mean (SEM). Differences were analyzed with either the Student’s *t*-test or the Kruskal-Wallis test. To determine statistical significance at different time points, we implemented one-way ANOVA. Correlation between tumor invasion and microglial accumulation or the level of brain slices was analyzed using a Spearman’s correlation test. *P*-values ≤ 0.05 were considered statistically significant. In the figures * represents *P* < 0.05; **P < 0.01; and ***P < 0.001).

## Results

### Brain Slices from Different Cortex Regions Demonstrate Comparable MCF-7 Cell Invasion

**Figure 1:**
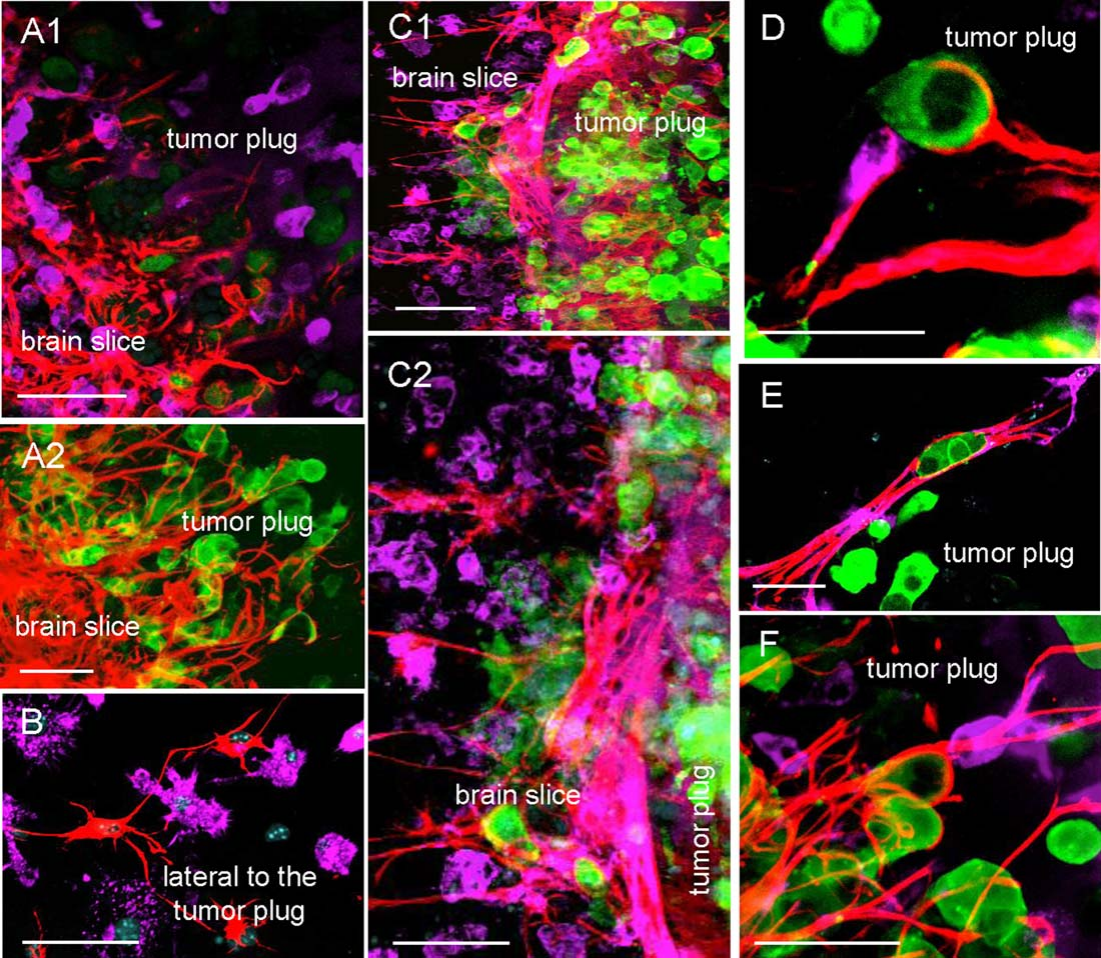
Astrocytes and microglia are part of gliosis and interact with MCF-7 in the slice cocultures. (A–F) Double staining of the coculture with the organotypic brain slice and MCF-7-GFP (green) in the 3D-tumor plug were applied for astrocytes (anti-GFAP-TRITC = red) and microglia (ILB4-Alexa Fluor 647 = violet), except (A2) is only a single astrocytic staining for better illustration (A1 and A2) The astrocytes form a mesh in the tumor plug contacting the MCF-7 cells with their protrusions. Interestingly, these astrocytes remain connected with other astrocytes and the brain tissue. (B) A lateral part of the brain slice which is adjacent to the tumor plug. In contrast to astrocytes, microglia leave the brain slice as individual cells (A1, B). In the brain tissue, both glial cells are activated at the invasion zone, especially the astrocytes attempt to form a dense barrier in the brain slice next to the tumor plug (C1 and the contact area in higher magnification C2). (D–F) These examples illustrate frequently detectable interactions of astrocytes and microglia with the same MCF-7 cells in the tumor plug. Scale bars represent 50 µm. [Color figure can be viewed in the online issue, which is available at wileyonlinelibrary.com.]

Since metastases occur in various regions of the brain, we first analyzed whether the region, from which the organotypic brain slice for our slice coculture was cut, had any influence on the degree of tumor cell invasion. Therefore, we categorized the cortex of the whole brain slices according to anatomy into three different cranial-caudal levels and performed cocultures adjacent to the cerebral cortex in the vicinity of the hippocampus (Supp. Info. Fig. 1A). There was no difference with regard to either the viability of the slice or the invasion of MCF-7 cells (Supp. Info. Fig. 1B,C).

### Astrocytes Are Part of the Glial Reaction

**Figure 2:**
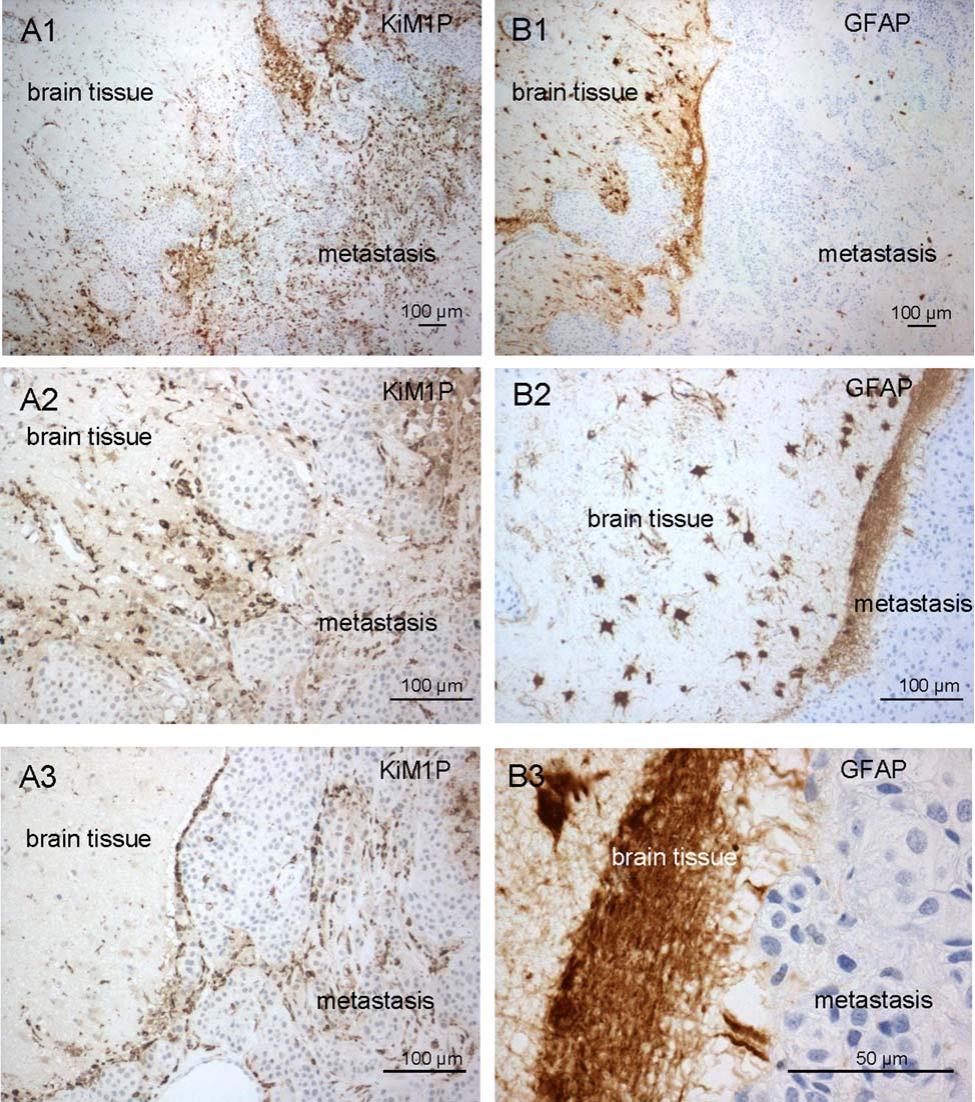
Distribution of microglia and astrocytes in human brain metastases. The reaction of the microglia and astrocytes in human brain metastasis samples is comparable to the brain slice coculture system. A1–A3) Macrophage/microglia staining was performed employing the anti-KiM1P antibody. Activated macrophages/microglia accumulate in the adjacent brain tissue and at the interface. The majority of the activated macrophages/microglia infiltrate into the metastatic tissue. (B1–B3) Astrocyte response was demonstrated by GFAP IHC staining. Activated astrocytes accumulate in the adjacent brain tissue and form a barrier at the interface to the metastatic tissue. Furthermore, comparable to the brain slice coculture system, only few cells enter the tumor mass. [Color figure can be viewed in the online issue, which is available at wileyonlinelibrary.com.]

Previously, it was shown that astrocytes are part of the gliosis reaction directly at the border with the metastatic tissue (Zhang and Olsson, 52). This led us to assume that astrocytes may be involved in the host tissue response against the growing metastatic and tissue destructing carcinoma cells. We compared the astrocyte and microglia reaction in brain slice coculture system by confocal investigations (Fig. 1) with human brain metastasis samples using immunohistochemistry (IHC) (Fig. 2). The confocal investigations of the 350-µm-thick whole brain slices revealed that astrocytes form direct contacts with the breast cancer cells at the interface with the 3D-plug and at the invasion front. However, astrocytes are organized as a mesh in the 3D-MCF-7 plug (Fig. 1A,B) and form a barrier in the brain tissue (Fig. 1C), whereas microglia act more heterogeneously and with the potential to leave the brain slice as individual cells and migrate into the 3D-plug. Microglia activation in particular is locally restricted to the contact area (see Supp. Info. video 1). Interestingly, both glial cells often contact and interact with the carcinoma cells, sometimes also simultaneously (Fig. 1D–F).

### Similar Morphology Observed in Human Brain Metastasis Samples

The glial reaction in the human brain metastasis samples is very similar to the slice coculture system in Fig. 1. Microglia/macrophages are also activated in the adjacent brain tissue and accumulate at the interface. Moreover, the reaction of microglia/macrophages at the interface as well as adjacent brain tissue is much severe when infiltrating carcinoma cells are present. This is demonstrated in Fig. 2A2, with infiltrating breast carcinoma cohorts, in comparison to Fig. 2A3, with a sharp border between the metastatic and the adjacent brain tissue. Moreover, in contrast to the astrocytes, the majority of microglia/macrophages infiltrate into the metastatic tissue (Fig. 2A1–3). The GFAP staining revealed the majority of the activated astrocytes in the adjacent brain tissue. Furthermore, they form a barrier at the interface to the metastatic tissue; and only few cells enter the tumor mass (Fig. 2B1–3). However, at the interface we detected astrocyte protrusions in direct contact with the carcinoma cells.

### Microglia-Assisted Invasion Is Restricted to Malignant Cells

**Figure 3:**
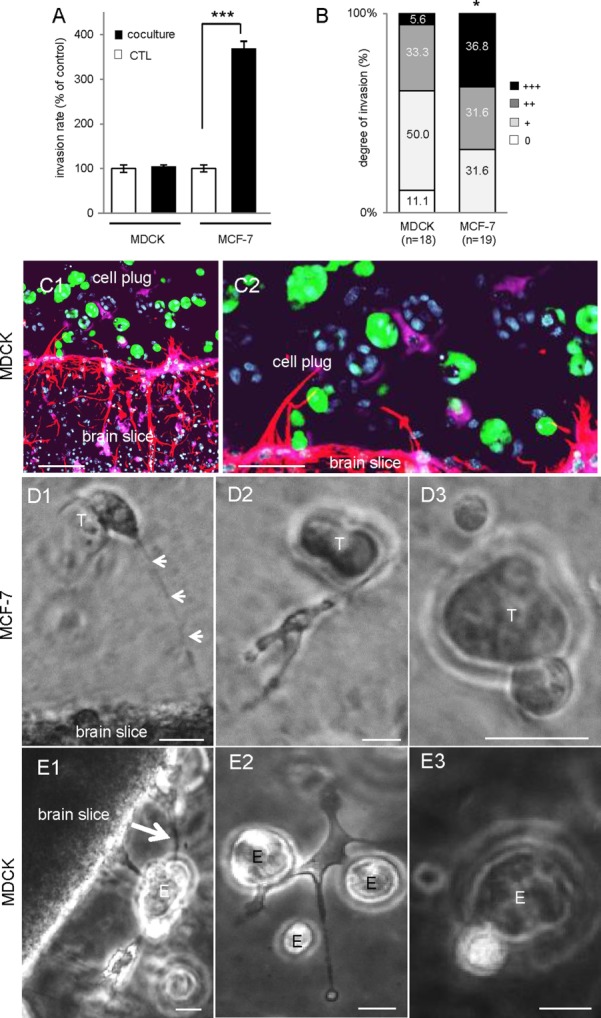
Comparison of the astrocytic and microglial reactions as well as their effects on benign and malignant epithelial cells. (A) Boyden chamber assays were performed with malignant MCF-7 or epithelial (benign) MDCK cells ± indirect microglia coculture. Data represent mean ± SEM with *n* ≥ 9. (B) Cocultures with 3D-MCF-7-GFP or 3D-MDCK-GFP cells. Data represent the degree of MCF-7 or MDCK invasion into the brain slice at the contact area after 96 h. (C) Double stainings of astrocytes (anti-GFAP-TRITC = red) and microglia (ILB4-Alexa Fluor 647 = violet) of the MDCK-GFP (green) coculture. The gliosis reaction of astrocytes and microglia was comparable to the MCF-7 coculture (see Fig. 1D), however, MDCK did not invade the slice (scale bars represent 50 µm). (D and E) Excerpts of time-lapse experiments illustrating three different microglial subtypes in MCF-7 and MDCK cocultures (T = MCF-7 cells; E = MDCK cells, white arrows = protrusions). Subtype A: microglia resident in the brain slice with long protrusions into the 3D-plug (D1 and E1). Subtype B: individual microglia in the plug forming short contacts (D2 and E2). Subtype C: round microglia directly attach to epithelial cell surface over a long period (D3 and E3). This kind of microglia sometimes transports both epithelial cell types (scale bars represent 20 µm). [Color figure can be viewed in the online issue, which is available at wileyonlinelibrary.com.]

Next, we inquired as to whether this gliosis reaction and microglia-induced invasion in our coculture system represents a specific phenomenon of malignant cells or whether it occurs in a nonselective manner. To this aim, we first measured the degree of invasion of the benign epithelial cell line MDCK as well as that of the malignant MCF-7 in a modified Boyden chamber assay with and without indirect coculture with microglia. In contrast to the carcinoma cells, the invasion of MDCK was not enhanced by microglia (*n* ≥ 9; *P* < 0.001) (Fig. 3A). Similarly, there was only minimal invasion of MDCK in the organotypic whole brain slice coculture, when compared with MCF-7 (*P* < 0.05) (Fig. 3B). Despite the diverging effects on invasion, the extent and morphology of the glial reaction did not differ between benign and malignant cells (Figs. 1C and 3C; Supp. Info. video 2 (MCF-7 coculture) and video 3 (MDCK coculture)). Using time-lapse microscopy, microglia-epithelial cell interactions could be further discriminated into at least three morphological subtypes in both coculture systems (Fig. 3D,E). We observed microglia reaching out from the brain slice with long protrusions (up to 120 µm) to contact individual epithelial cells as well as cell cohorts, pulling them toward the brain slice (subtype A). Microglia cells could also completely enter the 3D-cell plug. The subtype B cells had an elongated shape with long protrusions, were very mobile, and formed short-term contacts with both epithelial cell types. Subtype C cells were round and often attached to the surface of epithelial cells, sometimes acting as transporters as previously described (Pukrop et al., 37). In some cases, we detected a rapid transition between the latter two subtypes, indicating that microglia could switch between these phenotypes (see Supp. Info. video 4).

### MDCK But Not MCF-7 Cells Undergo Apoptosis in the Vicinity of the Brain Slice

**Figure 4:**
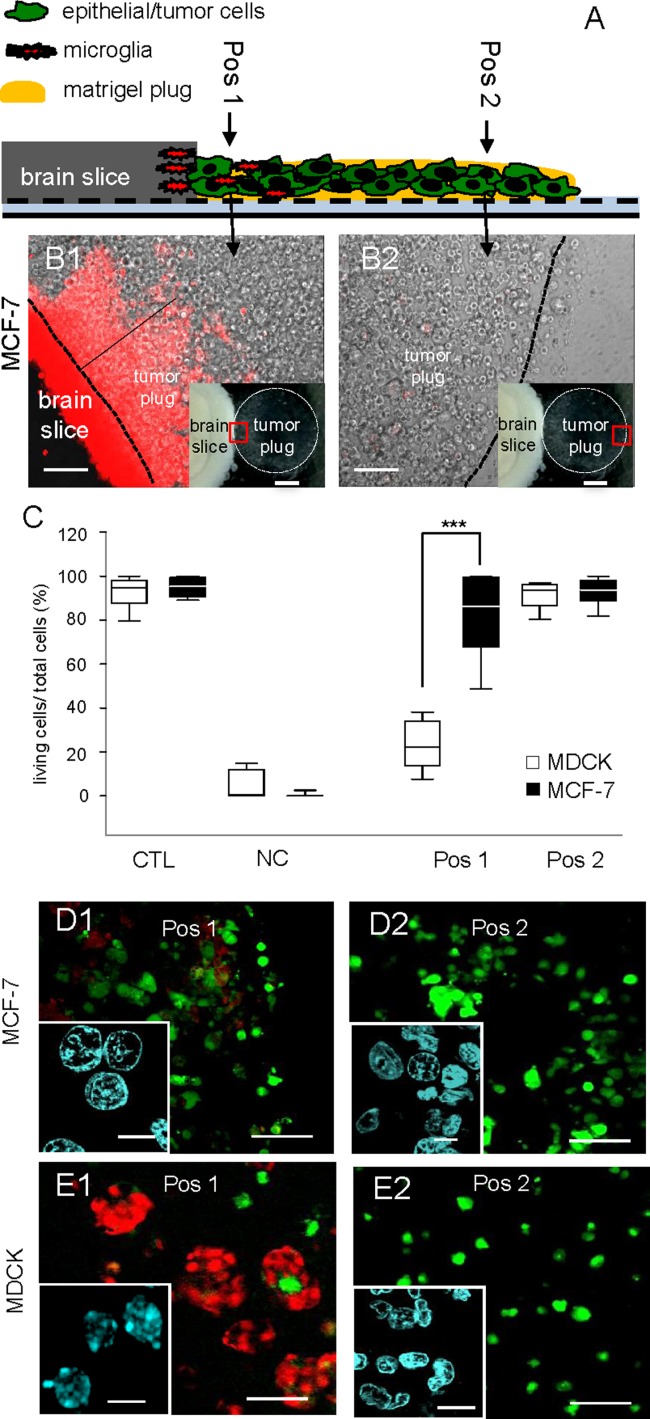
Astrocyte and microglia interaction leads to MDCK apoptosis while MCF-7 survives. (A) Scheme of the experimental procedures: the Position 1 (Pos 1) is directly adjacent to the brain slice; Position 2 (Pos 2) is at the contralateral side of the plug. (B) Representative phase contrast images of coculture time-lapse experiments with live microglial staining (red = ILB4-Alexa Fluor 568). At Pos 1 (B1), microglia leave the brain slice and distance themselves with a maximum of 600–800 µm from the brain slice edge, thus microglia are absent at Pos 2 (B2) (scale bars represent 200 µm). Inlays: the red rectangles illustrate the positions in the tumor plug (scale bars represent 1 mm). (C) Image J quantification: the box plots indicate the percentage of living cells compared with total cells. Monocultured cell/tumor plugs served as positive controls (CTL) and UV-irradiated cell/tumor plugs served as negative controls (NC). At Pos 1 MDCK viability was reduced significantly, in contrast MCF-7 cells demonstrated no significant change toward CTL or both positions (data represent mean ± SEM with *n* ≥ 9). (D and E) Representative confocal images of the live (Calcein-AM = green) and dead (PI = red) staining of the epithelial cell/tumor plugs (scale bars represent 100 µm), including the nuclei morphology (DAPI = blue, in the insert, scale bars represent 10 µm) were shown. [Color figure can be viewed in the online issue, which is available at wileyonlinelibrary.com.]

To further investigate why only malignant cells invade the brain although both cell types are contacted by glial cells in the same way at Position 1 (interface, for scheme refer to Fig. 4A) we analyzed MCF-7 and MDCK in the 3D-plug by live–dead staining after 96 h of coculture. At this point, we measured the viability at Position 1, the area at which glial-epithelial cell interactions take place, as well as in the opposite region (Position 2), at which these interactions are absent (Fig. 4B and Supp. Info. video 5). There was no significant difference in the viability of MDCK or MCF-7 cells at Position 2. However, the majority of the MDCK cells at Position 1 were positive for PI. This was in direct contrast to both the situation noted at Position 2 and to MCF-7 cells in general. To validate this distinction, we quantified the fluorescence signals in the double staining for live (calcein-AM, green) and dead cells (PI, red) using Image J software. We confirmed that only 22.3% (median) of the MDCK cells were still viable at Position 1, in comparison with 93.6% at Position 2 (Fig. 4C–E). Moreover, the MDCK nuclei at Position 1 demonstrated the typical features of apoptosis in contrast to Position 2 or to MCF-7 at both positions (Fig. 4D,E inserts). In summary, in the area of contact with the 3D-epithelial cell plug, the locally restricted glial reaction occurred independently of the nature of the epithelial cell. After the uniform contact phase, however, the glial reaction induces apoptosis in the benign epithelial cells. This fails to occur in carcinoma cells, with the added twist that the reaction actually appears to foster invasion.

### Microglia and WNT Are Involved in MDCK Apoptosis

**Figure 5:**
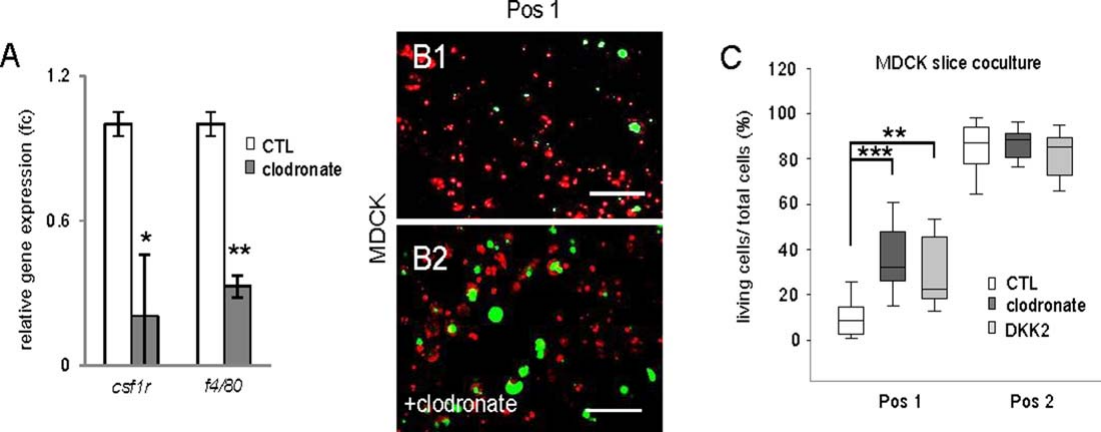
Microglia depletion and addition of DKK2 partially rescue MDCK apoptosis. (A) Clodronate (100 µg/mL) effectively reduced the microglial markers csf1r and f4/80 in whole brain slices after 48 h of stimulation. Gene expression levels were normalized to the average of the respective housekeeping genes and expressed as fold changes (fc). Data represent mean ± SEM with *n* ≥ 9 of three individual experiments. (B1 and B2) Representative confocal images of the live (Calcein-AM = green) and dead (PI = red) staining of the epithelial MDCK cells at Position 1. Microglia depletion leads to a partial rescue of MDCK viability after clodronate treatment (scale bars represent 100 µm). (C) Image J quantification: Clodronate or DKK2 treatment resulted in a partial rescue of the glia-induced MDCK apoptosis at Position 1. Data represent mean ± SEM with *n* ≥ 9 of three individual experiments. [Color figure can be viewed in the online issue, which is available at wileyonlinelibrary.com.]

Next, we addressed the question as to whether microglia are involved in MDCK apoptosis. We proceeded by depleting microglia in the organotypic brain slice using the bisphosphonate clodronate, as recently described (Kreutz et al., 26; Pukrop et al., 37). The microglial marker gene expression of *csf1r* and *f4/80* was significantly reduced in the entire brain slices after 48 h as determined by qRT-PCR (Fig. 5A). Although we already noted a slight but significant decrease in MDCK cell viability upon application of clodronate (100 µg/mL) in the control culture (Supp. Info. Fig. 2A), the depletion of microglia led to a significant increase in the number of living MDCK cells at Positon 1 in our coculture system (Fig. 5B,C). To test whether WNT-signaling, which is involved in microglia-induced carcinoma invasion (Pukrop et al., 37), also regulates microglia-induced apoptosis, we treated the MDCK-organotypic brain slice coculture with DKK2. This led to an increase of MDCK viability at Position 1 similar to that obtained with microglia depletion (Fig. 5C). Nevertheless, in contrast to microglia-induced MCF-7 invasion, which is almost completely abolished by these inhibitors (Pukrop et al., 37), both treatments could only achieve a partial rescue of apoptosis. This appears to indicate the involvement of at least one additional cell type, such as astrocytes. More important, microglia as well as WNT signaling seem to have more influence on transport and invasion of the cacrinoma cells than to apoptosis of the begnin epithelial cells.

### CXCR4 Is Involved in Microglia- and Astrocyte-Induced Invasion

**Figure 6:**
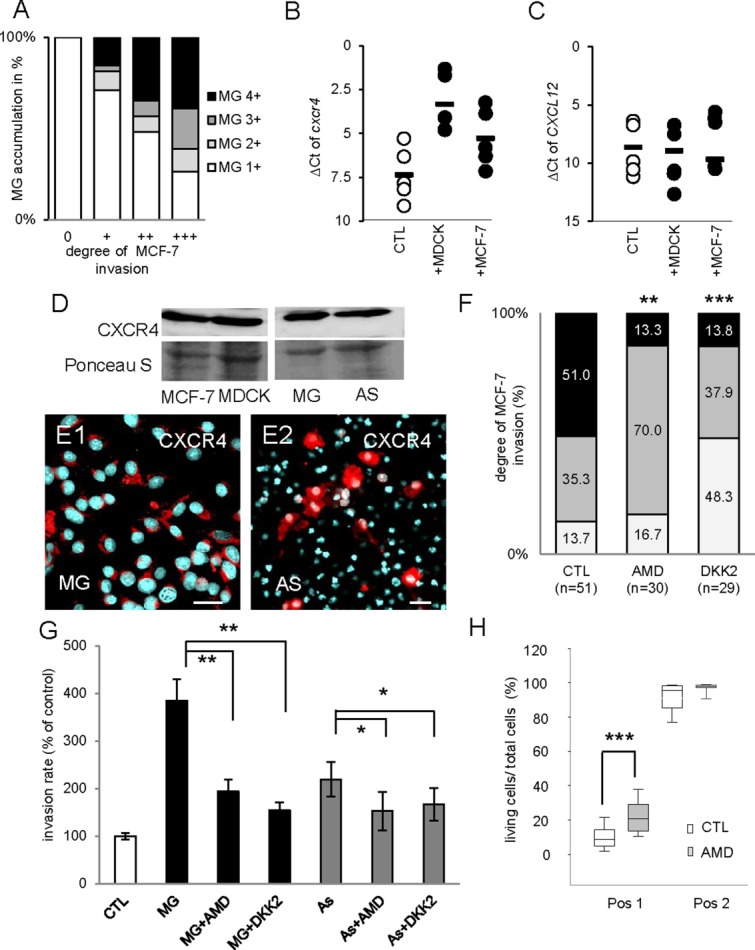
CXCR4 and WNT signaling are involved in microglia- and astrocyte-induced invasion. (A) The degree of MCF-7 invasion and microglia (MG) accumulation at the contact area/invasion front was determined in brain slice cocultures (*n* = 203). The analysis revealed a significant correlation between the degree of tumor invasion and the degree of microglial accumulation (Spearman’s test; *P* <  0.001). (B and C) qRT-PCR results of cxcr4 and cxcl12 illustrated by the ΔCt method. Coculture with MDCK or MCF-7 cells for 96 h upregulates cxcr4 but not its ligand, cxcl12 in the organotypic brain slices. Line represents the mean value of all experiments and each dot represents the mean value of triplicate analyses with *n* ≥ 15 from three individual experiments. (D) MDCK, MCF-7, microglia, and astrocytes (AS) express CXCR4 protein detected by immunoblot. (E1 and E2) The glial cells astrocytes and microglia reveal a heterogeneous expression pattern of CXCR4 (red) on immunofluorescence staining counterstained with DAPI (scale bars represent 20 µm). (F) The invasion of the MCF-7-GFP cells into the whole brain slice was significantly reduced after DKK2 as well as AMD3100 treatment. (G) The influence of indirect cocultures with microglia or astrocytes on carcinoma cell invasion measured by modified Boyden chamber assays. Treatment with WNT inhibitor DKK2 as well as CXCR4 inhibitor AMD3100 significantly reduced both the astrocyte- or microglia-induced carcinoma cell invasion. Data represent mean ± SEM with *n* ≥ 9 of three individual experiments. (H) The CXCR4 inhibitor also partially rescued the glial-induced apoptosis of MDCK at Position 1 (scheme see Fig. 4A) similar to clodronate or DKK2 treatment shown in Fig. 5C. Data represent mean ± SEM with *n* ≥ 9 from more than three individual experiments. [Color figure can be viewed in the online issue, which is available at wileyonlinelibrary.com.]

Considering the severe effects of microglia on malignant invasion as well as the link of microglia activation and infiltrating carcinoma cells (as demonstrated above Fig. 2A2–3), we investigated the correlation between astrocytes/microglia accumulation in the contact area and the degree of MCF-7 invasion in our coculture system. While the degree of astrocyte accumulation did not have any major influence, microglia accumulation significantly correlated with MCF-7 invasion based on a series of 203 coculture experiments (*P* < 0.001) (Fig. 6A). We further evaluate the candidate genes which are possibly involved in microglia-induced by qRT-PCR. Consistent with our previous microarray results on microglia cells (Pukrop et al., 37), *cxcr4* but not its ligand *cxcl12* (also known as stromal cell-derived factor-1, *SDF1*) was upregulated in brain slices upon coculture (Fig. 6B,C). We then scrutinized the protein expression of CXCR4 in MDCK, MCF-7, microglia, and astrocyte. All cell types expressed CXCR4 (Fig. 6D). Interestingly, both epithelial cell types depicted rather homogeneous staining (Supp. Info. Fig. 3), whereas microglia and astrocyte revealed a heterogeneous expression pattern (Fig. 6E). We then analyzed the influence of murine astrocytes on carcinoma cell invasion in comparison with microglia-induced invasion. Astrocyte coculture also induced carcinoma cell invasion in modified Boyden chamber assays, however, to a much lower extent than microglia (Fig. 6G). Additionally, both kinds of glia-induced cancer cell invasion were significantly inhibited by the WNT-inhibitor DKK2 as well as by AMD3100, a CXCR4 inhibitor (Fig. 6G) without cytotoxicity either to the epithelial cells, MCF-7 and MDCK, or the glia cells, microglia and astrocytes (Supp. Info. Fig. 2B). Both inhibitors also reduced invasion of MCF-7 into the whole brain slice to almost the same extent (Fig. 6F). In contrast, the viability of MDCK at Position 1 was only slightly increased by the CXCR4 inhibitor, again comparable to DKK2 treatment and microglia depletion (Fig. 6H). In conclusion, microglia have less influence on MDCK apoptosis than on invasion of the carcinoma cells, for which the effects of WNT or CXCR4 inhibition as well as microglia depletion are more pronounced.

### CXCR4 Expression in Astrocytes and Microglia in Brain Metastasis Samples

**Figure 7:**
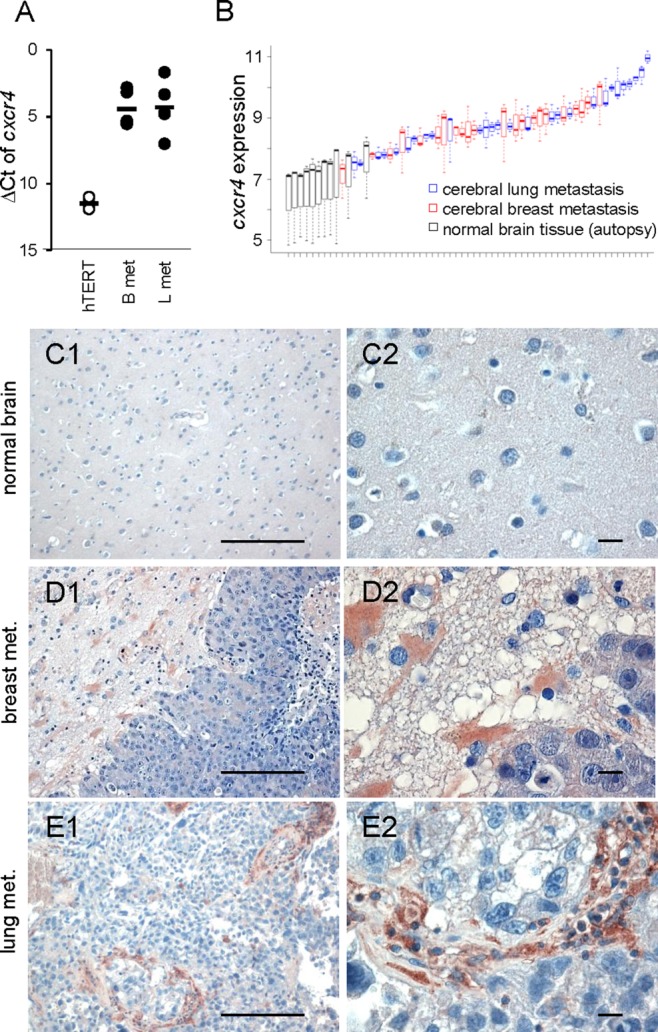
CXCR4 expression in astrocytes and microglia in human brain metastasis samples. (A) qRT-PCR results of cxcr4 illustrated by the ΔCt method. Human cerebral lung (L met) and breast cancer (B met) metastases samples demonstrate a marked expression of CXCR4 in contrast to the benign epithelial cell line hTERT. Line represents the mean and each dot represents the mean value of triplicate analyses with *n* ≥ 15. (B) Gene expression of cxcr4 is visualized using boxplots across all samples for the three different cxcr4 probes (209201_x_at, 217028_at, 211919_s_at). Samples are ordered by means of cxcr4 gene expression. Black: normal brain tissues, red: cerebral breast metastases, and blue: cerebral lung metastases. (C–E) Expression and localization of CXCR4 were analyzed in normal brains, cerebral metastasis of breast and lung cancer samples by immunohistochemistry. In normal brains, CXCR4 was undetectable (C), whereas in cerebral metastases, CXCR4 expression was detectable in the metastatic cells, the metastatic stroma, and especially in astrocytes in the gliosis region adjacent to the tumor cells (D and E) (scale bars represent 20 µm, in the left panel 200 µm). [Color figure can be viewed in the online issue, which is available at wileyonlinelibrary.com.]

Since we detected remarkable expression of *cxcr4* in all of the five investigated human cerebral metastases of breast and lung carcinomas (Fig. 7A), we assessed the relevance of our qRT-PCR results in external gene sets, one from normal brain autopsies (GSE5389; *n* = 11), a second from breast (GSE14017, GSE14018; *n* = 22), and the third from lung adenocarcinoma brain metastasis (GSE14108; *n* = 28). As expected, the normal brain samples revealed the lowest expression, whereas *cxcr4* expression in lung and breast metastases demonstrated a wide range, including samples with very high expression levels (Fig. 7B). Interestingly, the ligand *cxcl12* was not upregulated in comparison to normal brain (Supp. Info. Fig. 4). Next, we analyzed the CXCR4 protein localization in normal brain tissue and cerebral metastases. First, our histological findings confirmed the gene expression pattern. While we detected no CXCR4 expression in normal tissue at all, the metastasis samples demonstrated an expression ranging from absence to very high levels. Most importantly, in samples with evaluable adjacent brain tissue, we discovered CXCR4 expression in the gliosis region next to the metastatic tissue in 80% of breast and 68.8% of lung cancer cerebral metastasis (Table1; Fig. 7C–E).

### CXCR4 Is Involved in Glial Response After Various Types of Brain Injury

**Figure 8:**
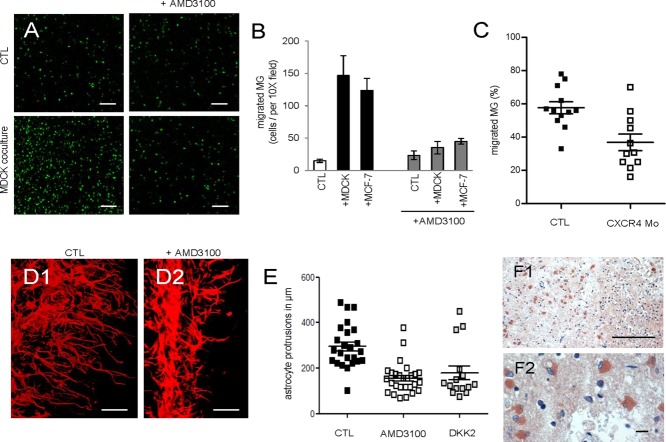
CXCR4 knockdown/ inhibition affects microglial and astrocytic damage response. (A and B) Results of the microglia chemotaxis assays toward MDCK and MCF-7 cells. Both cocultures significantly enhanced microglia migration. (A) Representative microglial images were shown ± MDCK coculture and ± treatment with the CXCR4 inhibitor (AMD 3100, 1 µg/mL). AMD3100 reduced the enhanced microglial migration toward MDCK almost to CTL level (scale bars represent 200 µm). (B) Results demonstrate migrated microglial cells per 10× field, data represent mean ± SEM; *n* = 40. (C) Knockdown effects of CXCR4 in zebrafish larvae: Percentage of microglia in the optic tectum moving to the injury site in CTL (*n* = 12) and CXCR4-morpholino (CXCR4-Mo) injected embryos (*n* = 11) were measured. The CXCR4-Mo significantly reduced the microglial migration toward the injury site (line = mean and SEM, *P* = 0.004). (D and E) Cocultured with 3D-MCF-7-GFP for 96 h ± 1 µg/mL of AMD3100 were stained with anti-GFAP-TRITC (red). The length of the astrocyte protrusions were reduced after AMD3100 (*P* <  0.001) or DKK2 (*P* = 0.009) treatment (scale bars represent 100 µm); line = mean and SEM (*n* ≥ 15). (F) Protein expression of CXCR4 in human stroke samples using IHC (scale bar represents 20 µm, in the upper panel 200 µm). [Color figure can be viewed in the online issue, which is available at wileyonlinelibrary.com.]

We then shifted our focus onto the influence of CXCR4 on microglia recruitment and chemotaxis. Migration of microglia toward MDCK and MCF-7 was significantly reduced by AMD3100 (Fig. 8A,B), again independently of the epithelial cell type. To clarify whether this is conferrable to other types of cerebral damage response, microglia recruitment was measured in a brain injury assay in the microglia transgenic zebrafish line (pu1:Gal4-UAS-GFP). These transgenic zebrafish allowed us to quantify *in vivo* the migration of individual microglia cells toward the brain injury. In control animals, 56% of all microglia cells were recruited to the site of injury. Following CXCR4-specific gene knock out by morpholino treatment, this was reduced to 30.7% (median; *P* = 0.004) (Fig. 8C). Interestingly, the effect was the more pronounced the larger the distance was between microglia and injury, supporting the hypothesis of a chemotactic gradient (Supp. Info. video 6).

With regard to CXCR4 expression on the astrocytes in culture and the human metastasis samples, we investigated the morphological changes after AMD3100 and DKK2 treatment in the brain slice coculture model. Both inhibitors had a similar influence on the length of astrocytic protrusions (Fig. 8D,E). Additionally, we performed CXCR4 IHC in stroke patients in whom we discovered an upregulation of CXCR4 in the glial cells and especially astroglial cells (Fig. 8G).

## Discussion

Our previous results demonstrated a comparable microglia reaction and assisted invasion for the human breast cancer cells MCF-7 as well as the murine breast cancer cell 410.4 (Pukrop et al., 37). As a proof of principle of a benign epithelial cell line, we chose the Madin-Darby Canine Kidney (MDCK) cells in direct comparison with the malignant MCF-7, originally derived from a metastatic patient. Other immortalized epithelial cell lines, like the epithelial breast cells hTERT, MCF-10A, and HMLE, were incompatible with the brain slice conditions.

As illustrated by time-lapse and confocal microscopy in the first phase, both glial cell types are activated and accumulate in the brain slice at the contact area with both epithelial cell types. In the second phase, astrocytes construct a close-knit meshwork directly at the interface, forming dense interactions with the potential intruders and finally embedding these in the meshwork. At the same time, microglial cells demonstrate different modes of duties and interactions supporting the recently emerging concept of responder subtypes of microglia (Scheffel et al., 43). Interestingly, some microglia detach themselves from the brain slice rim and enter the tumor plug up to a distance of about 800 µm. Consequently, interactions between epithelial cells and astrocytes as well as microglia mainly take place at the interface of the reactive brain tissue and the 3D-plug. Most importantly, the morphology of the coculture system reflects the human situation demonstrated by our and previous histological findings (Bos et al., 7; Fitzgerald et al., 13; Lorger and Felding-Habermann, 29; Pukrop et al., 37; Zhang and Olsson, 52). However, in the coculture system we are able to investigate the dynamics of the glia-carcinoma cell interaction, including bright field time-lapse imaging.

Thus our results propose that epithelial cells foreign to the brain are met by a general glial response of astrocytes as well as microglia. Importantly, this response is uniform in the first phase but with opposite outcome in the second phase. Benign cells, such as MDCK, are effectively killed by apoptosis, while the malignant MCF-7 or 410.4 are not eliminated. Not only do they survive this attack, they actually misuse these interactions to their advantage to foster their invasion.

Recently, it was additionally demonstrated that astrocytes could foster malignant cell proliferation and induce chemotherapy resistance in tumor cells (Kim et al., 23; Lin et al., 28; Seike et al., 45). Here we demonstrate that they may also enhance malignant invasion. Thus, astrocytes and microglia activation could influence different characteristics of malignant cells and could exacerbate cerebral metastasis and not only the course of neurodegenerative diseases.

In contrast to these tumor-promoting functions, it is also known that similar to our findings in the MDCK coculture, microglia together with astrocytes are capable of inducing apoptosis at least in neurons (Bezzi et al., 4). To what extent astrocytes contribute to the apoptosis of MDCK in our system needs further clarification. However, the coincidence that carcinoma cells are met by activated astrocytes and microglia directly after extravasation (Lorger and Felding-Habermann, 29) could be one explanation for the high range of carcinoma cell apoptosis in this step of cerebral metastasis (Chambers et al., 9; Kienast et al., 21).

Furthermore, it is generally accepted that circulating carcinoma cells disseminate to distant organs early in the often long-lasting process of successful metastasis. Avoiding destruction of carcinoma cells during the first steps of metastasis in the primary tumor or as circulating carcinoma cells by the immune system is one of the recently added hallmarks of cancer (Hanahan and Weinberg, 16). Considering that microglia together with astrocytes have the capacity to induce apoptosis of epithelial cells, we assume that resistance to the organ-specific responses against the intruders could be an additional important characteristic in the final steps of metastasis, the colonization. Early reports of liver metastasis support this theory (Gjoen et al., 15; Heuff et al., 18), in which the Kupffer cells were able to kill colon carcinoma cells effectively. Thus, the resistance to organ-specific responses directed toward the intruding epithelial cells could prove to be the decisive function for successful colonization by cancer cells.

At least two candidate pathways obviously play a role in this setting. We recently identified WNT as a key regulator of microglia-induced invasion (Pukrop et al., 37). Here we not only confirm this finding, we also reveal that the enhanced invasion by astrocytes is also responsive to the inhibition of WNT signaling by DKK2. Altogether, these findings underline the role of WNT signaling during cerebral colonization (Bleckmann et al., 6; Klemm et al., 25; Nguyen et al., 33; Pukrop et al., 37; Smid et al., 46).

Additionally, we demonstrate that the chemokine receptor CXCR4 is involved in astrocyte as well as microglia response in the coculture with the epithelial cells. It regulates the migration of the microglia and at least the length of the astrocyte protrusions. Most importantly, inhibition by AMD3100 reduces the glia-assisted invasion in all coculture settings. However, the analysis of the gene arrays and qRT-PCR demonstrates no upregulation of SDF1 in brain metastasis, whereas the receptor itself is. Metastatic growth causes a lot of cell and tissue damage, which leads to the release of ubiquitin to the extracellular space; thus ubiquitin could lead to the activation of the corresponding DAMP receptor CXCR4. That CXCR4 can act in damage response of microglia is illustrated by the zebrafish experiments and by the expression of CXCR4 in stroke patients as demonstrated here and by others (Salmaggi et al., 42; Wang et al., 51). Furthermore, CXCR4 signaling in microglia and astrocytes has been observed in the context of HIV-induced neuronal apoptosis and subsequent dementia (Bezzi et al., 4). Interestingly, we were also able to reduce partially the glia-induced apoptosis of MDCK by AMD3100. Finally, it should be noted that CXCR4 has physical—and most likely also functional—association with TLR4, the latter complex being more and more accepted for DAMP recognition (Triantafilou et al., 50). Thus, all these findings underline the role of CXCR4 in brain tissue damage response in both physiologic as well as pathologic situations, including cerebral metastasis.

However, CXCR4 and WNT signaling are involved predominantly in malignant progression but not so much in glia-induced apoptosis of benign epithelial cells. This indicates that these pathways are not the key players in glia-mediated cell killing. Obvious candidates like NO or TNFα for this effect will be tested in the MDCK coculture with inhibitors or neutralizing antibodies.

Taken together, the activation of at least one well-established pathway for repair and regeneration (WNT) and additionally the recently defined DAMP receptor CXCR4 links this glial activation to a damage response. This is further supported by the finding that the best-studied DAMP signaling system (TLR) was also under the most affected pathways in our previous screening in microglia. Moreover, the glial reaction and its signaling seems transferable to other injury models and diseases of the brain tissue. Thus, epithelial cells foreign to the brain induce a kind of damage response and induce the assembly of astrocytes and microglia at the interface, which is the prerequisite for the subsequent step of cell killing. In the case of malignant cells, however, this initial phase of damage-induced liaison is not followed by the phase of effective apoptosis, providing the malignant cells with the opportunity to exploit the glial contact to enhance their invasion. Although the exact mechanism as to how malignant cells escape apoptosis is unclear, both phases are obviously dependent on WNT and CXCR4 signaling, however with more effects on malignant progression than on glia-mediated apoptosis.

Finally, the manipulation of the organ-specific damage response may prove to be an innovative therapeutic strategy in the treatment or even prevention of cerebral metastasis. However, we have to consider that other host organs differ in their damage response. Therefore, we will really need to understand fully the damage systems and their responses in all affected organs after the processes of dissemination, during seeding, and growth of micro- as well as macrometastasis in the future.
