# Supplementary figures and images for: Carcinoma cells misuse the host tissue damage response to invade the brain
Source: Glia. 2013 Jul 6;61(8):1331–46. doi: 10.1002/glia.22518 (PMC3842117; doi:10.1002/glia.22518)

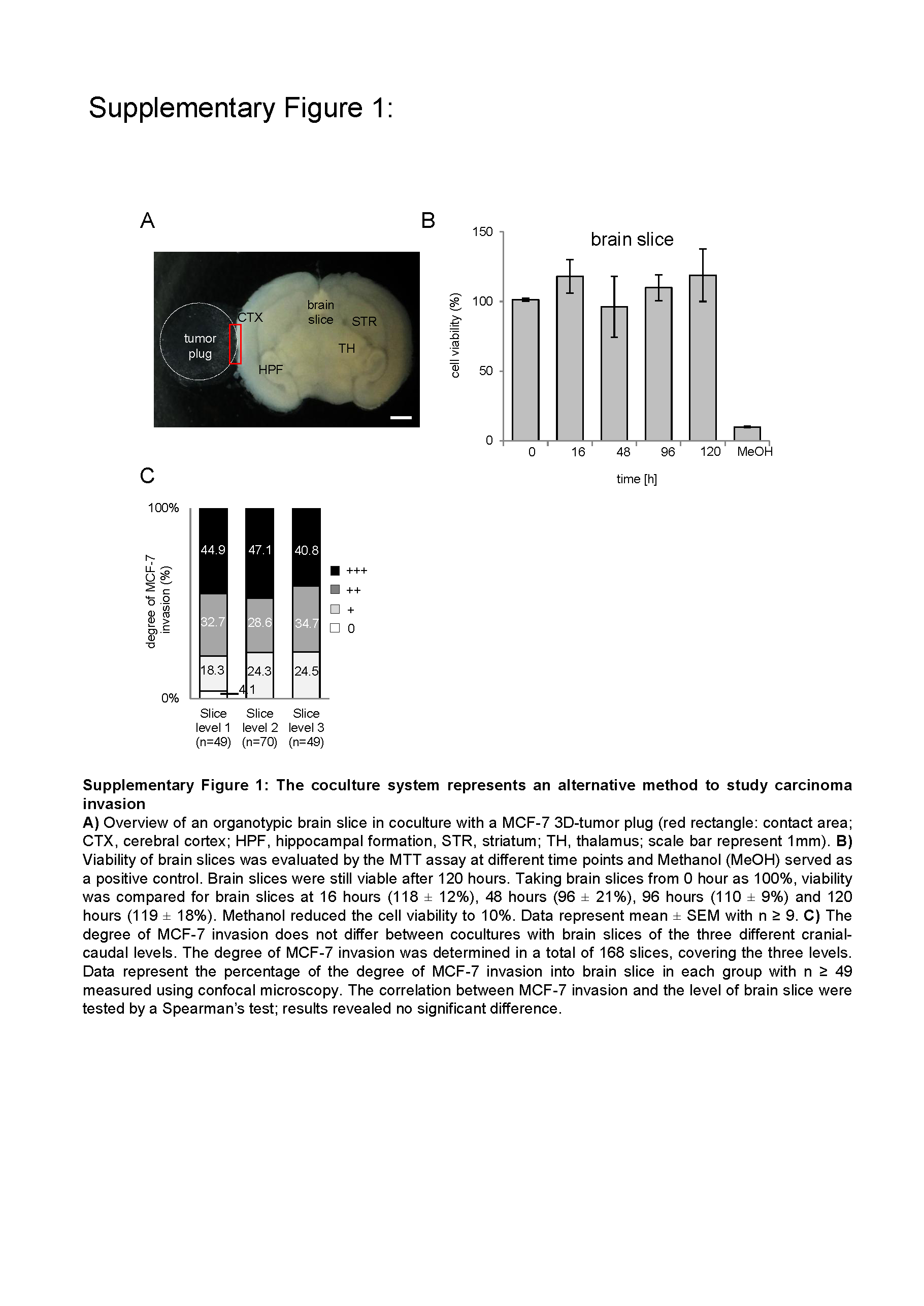

Supplement: Supplementary file 1 [file glia0061-1331-sd1.tif]

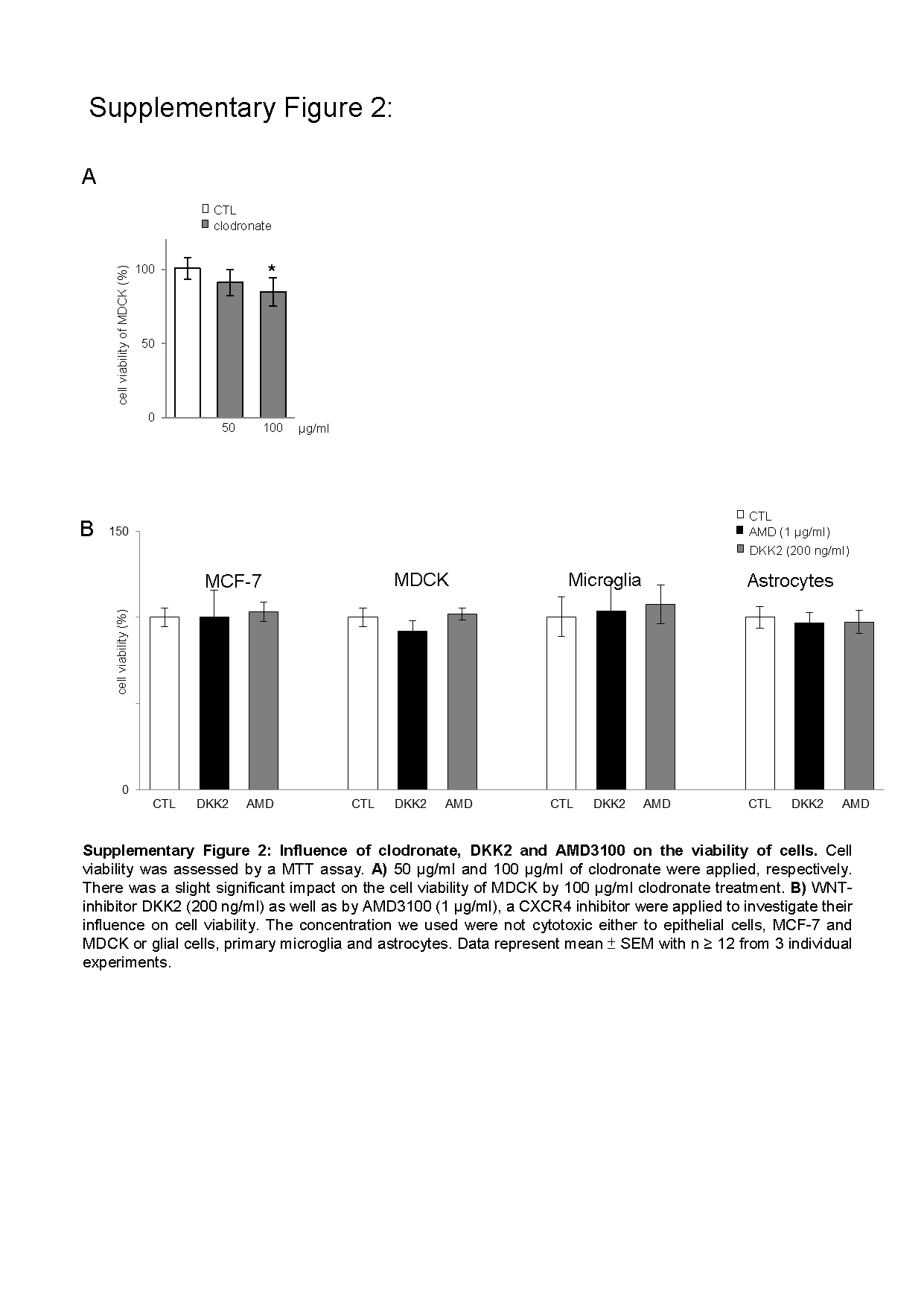

Supplement: Supplementary file 2 [file glia0061-1331-sd2.tif]

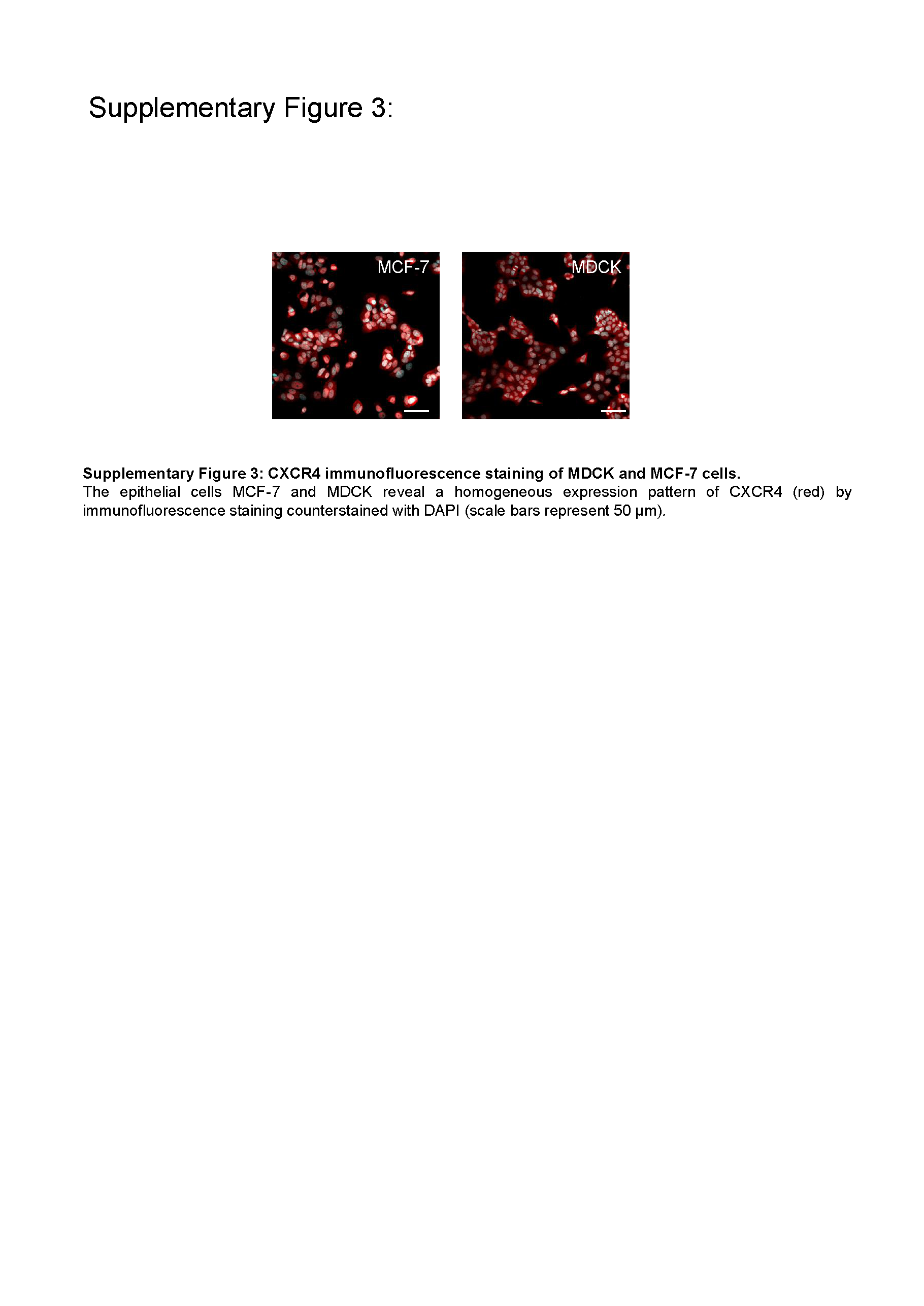

Supplement: Supplementary file 3 [file glia0061-1331-sd3.tif]

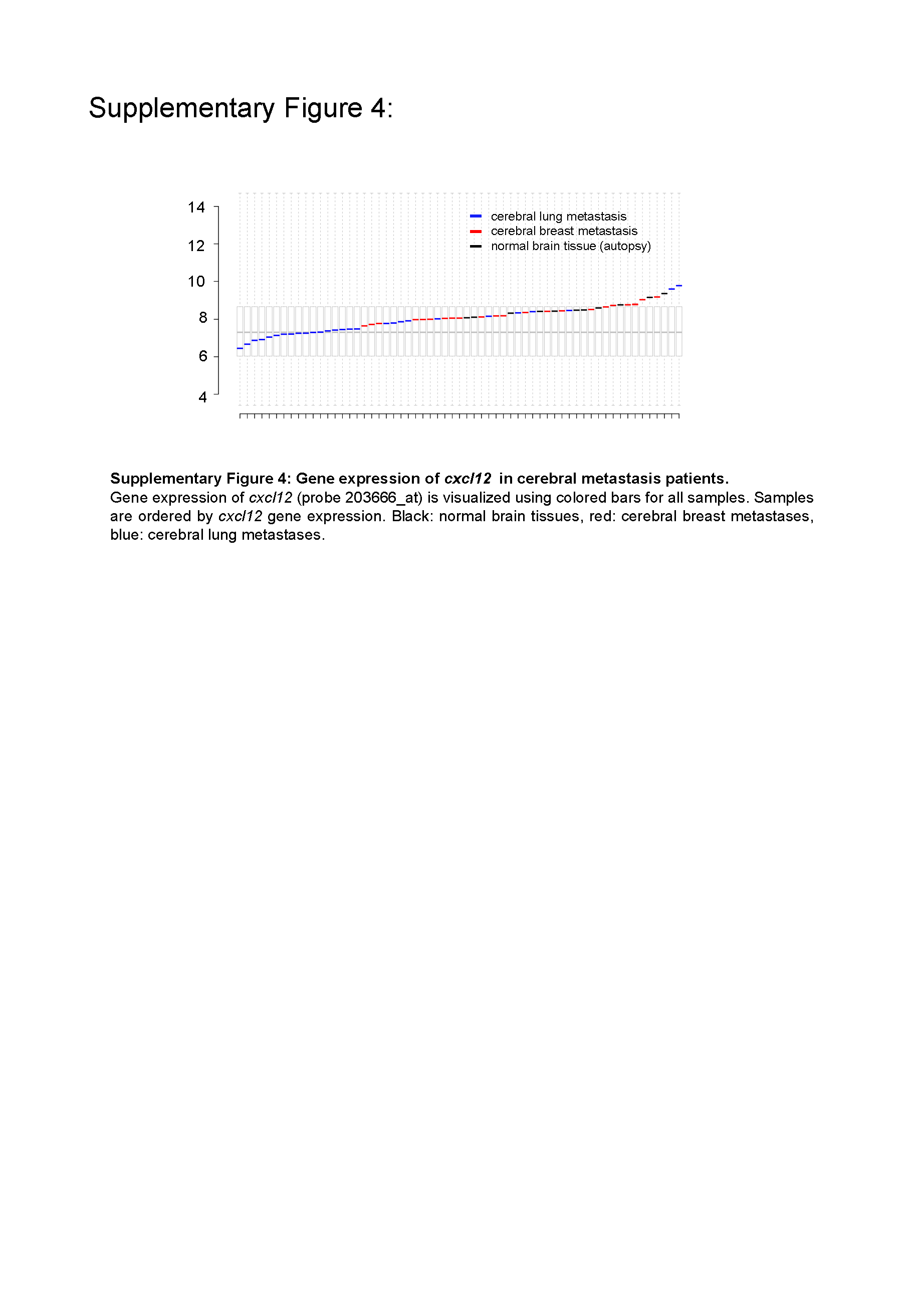

Supplement: Supplementary file 4 [file glia0061-1331-sd4.tif]
