# Supplementary material for: Carcinoma cells misuse the host tissue damage response to invade the brain
Source: Glia. 2013 Jul 6;61(8):1331–46. doi: 10.1002/glia.22518 (PMC3842117; doi:10.1002/glia.22518)
Supplement: Supplementary file 5 [file glia0061-1331-sd5.doc]

Supplementary Table 1

| **Gene** | **Forward Primer (5🡪3)** | **Reverse Primer (5🡪3)** | **Amplicon Size (bp)** | **Reference** |
| --- | --- | --- | --- | --- |
| **mouse primers** |  |  |  |  |
| *csf1r* | GCATACAGCATTACAACTGGACCTACC | CAGGACATCAGAGCCATTCACAG | 256 | 1 |
| *f4/80* | CGCTGCTGGTTGAATACAGAGA | CGGTGAGCAGACAGTGAATGA | 325 |  |
| *cxcr4* | GTGCAGCAGGTAGCAGTGAA | TTGCCGACTATGCCAGTCAAG | 248 |  |
| *pgk1 (HK)* | GAAGGGAAGGGAAAAGATGC | GCTATGGGCTCGGTGTGC | 137 | 2 |
| *18S rRNA (HK)* | GTAACCCGTTGAACCCCATT | CCATCCAATCGGTAGTAGCG | 151 | 3 |
| **human primers** |  |  |  |  |
| *cxcr4* | CCTATGCAAGGCAGTCCATGT | GGTAGCGGTCCAGACTGATGA | 86 |  |
| *cxcl12* | CTCAACACTCCAAACTGTGC | GTTTAAAGCTTTCTCCAGGTACTC | 117 |  |
| *hprt1 (HK)* | TGACACTGGCAAAACAATGCA | GGTCCTTTTCACCAGCAAGCT | 93 | 4 |
| *gnb2l1 (HK)* | GAGTGTGGCCTTCTCCTCTG | GCTTGCAGTTAGCCAGGTTC | 224 | 5 |

**Primer sequences**

*Csf1R* primer for mouse , *pgk1* primer for mouse, , *hprt1* for human, *gnb2l1* for human .

**Abbreviations**

*csf1r* : colony stimulating factor-1 receptor

*cxcr4* : C-X-C chemokine receptor type 4

cxcl12: chemokine (C-X-C motif) ligand 12

*hprt1*: hypoxanthine phosphoribosyltransferase 1

*gnb2l1*: guanine nucleotide binding protein, beta polypeptide 2-like 1

**1**.Kronsbein HC, Jastorff AM, Maccarrone G, Stalla G, Wurst W, Holsboer F, Turck CW, Deussing JM (2008) CRHR1-dependent effects on protein expression and posttranslational modification in AtT-20 cells. *Molecular and Cellular Endocrinology* 292: 1-10

**2**.Schmittgen TD, Zakrajsek BA (2000) Effect of experimental treatment on housekeeping gene expression: validation by real-time, quantitative RT-PCR. *J Biochem Biophys Methods* 46: 69-81

**3**.Vandesompele J, De Preter K, Pattyn F, Poppe B, Van Roy N, De Paepe A, Speleman F (2002) Accurate normalization of real-time quantitative RT-PCR data by geometric averaging of multiple internal control genes. *Genome Biol* 3: RESEARCH0034

**4**.Weisberg SP, McCann D, Desai M, Rosenbaum M, Leibel RL, Ferrante AW, Jr. (2003) Obesity is associated with macrophage accumulation in adipose tissue. *J Clin Invest* 112: 1796-1808

**5**.Zhang X, Ding L, Sandford AJ (2005) Selection of reference genes for gene expression studies in human neutrophils by real-time PCR. *BMC Mol Biol* 6: 4

**Supplementary Video 1**

**Microglial reaction is locally restricted to a distinct area next to the 3D-cell plug**

Time-lapse microscopy of organotypic brain slice-MCF-7 cocultures. In order to visualize microglia (MG), organotypic brain slice-MCF-7 cocultures were prestained with ILB4-Alexa Fluor 568 before imaging (red = MG; scale bars represent 200 µm). **Part 1**: Microglial reactions and movements *in the brain slice* at the contact area (t = 16-57 hours of coculture). **Part 2**: Microglial reaction and movements *in the periphery* of the contact area (t = 16-57 hours of coculture).

**Supplementary Video 2**

**Glial interactions in the organotypic brain slice-MCF-7 coculture at the contact area**

Bright field time-lapse microscopy of organotypic brain slice-MCF-7 cocultures was implemented at the contact area (at Position 1, see Figure 3A). Part 1.1: Overview of organotypic brain slice and 3D-MCF-7 plug coculture (t = 46-94 hours of coculture; scale bars represent 100 µm). Part 1.2: Higher magnification (t = 29-56 hours of coculture; scale bars represent 50 µm).

**Supplementary Video 3**

**Glial interactions in the organotypic brain slice-MDCK coculture at the contact area**

Bright field time-lapse microscopy of organotypic brain slice-MDCK cocultures was applied in the vicinity of the brain slice (at position 1, see Figure 3A). Part 1.1: Overview of organotypic brain slice and 3D-MDCK plug coculture (t = 76-107 hours of coculture; scale bars represent 100 µm). Part 1.2: Higher magnification (t = 52-63 hours of coculture; scale bars represent 50 µm).

**Supplementary Video 4**

**Different subtypes of Microglia-MCF-7 interactions**

In order to visualize microglia, organotypic brain slice-MCF-7-GFP (green) cocultures were prestained with ILB4-Alexa Fluor 568 before imaging (MG = red). The three noted morphological subtypes of microglia are displayed (see also Figure 2D and E). **Part 1**: Subtypes A-C of microglia (t = 53-70 hours of coculture, scale bars represent 50 µm). **Part 2:** Subtype C serve as a transporter of the MCF-7-GFP to the brain slice edge (t = 53-62 hours of coculture, scale bars represent 50 µm).

**Supplementary Video 5**

**Microglia are absent at Position 2 in the 3D-cell plug**

Time-lapse microscopy of whole brain organotypic slice cocultures in the opposite region of the 3D-MCF-7 plug. Images were recorded at Position 2 (see Figure 3A). Microglia were prestained with ILB4-Alexa Fluor 568 before live imaging. At Position 2, microglia were absent (t = 16-62 hours of coculture; red= MG; scale bars represent 200 µm).

**Supplementary Video 6**

**CXCR4 knockdown reduced microglial migration towards brain lesions in zebrafish.**

Three-day-postfertilization (3dpf) zebrafish larvae were used and microglia were visualized using the pu1:Gal4-UAS-GFP transgenic line (green = MG). Zebrafish embryos with or without CXCR4 morpholino oligonucleotide injection were applied for live imaging. Lesions were generated using a pulsed 532 nm laser coupled to the FV1000. **Part 1**: CTL (t = 0-2 hours). **Part 2**: CXCR4 morpholino group (t = 0-2 hours). Scale bars represent 20 µm.
